# Sitagliptin and Fractures in Type 2 Diabetes: A Nationwide Population-Based Propensity-Matching Study

**DOI:** 10.3389/fphar.2018.00677

**Published:** 2018-06-22

**Authors:** Shih-Yi Lin, Wu-Huei Hsu, Cheng-Chieh Lin, Cheng-Li Lin, Chun-Hao Tsai, Hung-Chieh Yeh, Chung-Y. Hsu, Chia-Hung Kao

**Affiliations:** ^1^Graduate Institute of Clinical Medical Science, College of Medicine, China Medical University, Taichung, Taiwan; ^2^Division of Nephrology and Kidney Institute, China Medical University Hospital, Taichung, Taiwan; ^3^Division of Pulmonary and Critical Care Medicine, China Medical University Hospital and China Medical University, Taichung, Taiwan; ^4^Department of Family Medicine, China Medical University Hospital, Taichung, Taiwan; ^5^Management Office for Health Data, China Medical University Hospital, Taichung, Taiwan; ^6^College of Medicine, China Medical University, Taichung, Taiwan; ^7^Department of Orthopedics, China Medical University Hospital, Taichung, Taiwan; ^8^Graduate Institute of Clinical Medical Science, China Medical University, Taichung, Taiwan; ^9^Department of Nuclear Medicine, China Medical University Hospital, Taichung, Taiwan; ^10^Department of Bioinformatics and Medical Engineering, Asia University, Taichung, Taiwan

**Keywords:** fracture, sitagliptin, dipeptidyl peptidase-4 inhibitor, cohort study, diabetes

## Abstract

**Background:** Sitagliptin, a dipeptidyl peptidase-4 inhibitor possibly affects bone turnover. We conducted this cohort study to determine whether sitagliptin is associated with an increased risk of fracture.

**Methods:** The sitagliptin cohort included 1,578 patients aged 20 years and above. The nonsitagliptin cohort comprised propensity-score matched patients at a ratio of 1:1. The primary outcome was the incidence of fractures, which was evaluated using Kaplan–Meier survival analysis and proportional hazards modeling.

**Results:** The mean age of patients in the sitagliptin and nonsitagliptin cohorts was 63.1 and 63.3 years, respectively. The incidence of fractures in the sitagliptin cohort was 46 per 1,000 person-years and that in the nonsitagliptin cohort was 40.8 per 1,000 person-years. Compared with patients in the nonsitagliptin cohort, those in the sitagliptin cohort who received sitagliptin for ≥250 days had a higher risk of fracture (aHR = 1.32, 95% CI = 1.06–1.64).

**Conclusion:** Using sitaglipin ≥250 days was associated with an increased risk of fracture.

## Introduction

Fracture, most often occurring in the hip, is identified as a complication of type 1 and 2 diabetes (Forsén et al., 1999). With economic growth, changing dietary patterns, and relatively reduced insulin secretory function, the age-standardized prevalence of diabetes in adults has been increasing, particularly in Asian and other developing countries (Rhee, 2015; NCD Risk Factor Collaboration (NCD-RisC), 2016). Strotmeyer et al reported old age as the most significant risk factor for fractures in patients with diabetes (Strotmeyer et al., 2005). Fractures caused substantial morbidity and mortality in elderly people, resulting in the frequent necessity for long-term care (Manton et al., 1997). In addition to population growth and aging, such an increase in the incidence of fractures has led to the awareness of fracture prevention in elderly populations, particularly high risk groups having diabetes (Gonnelli et al., 2015).

In addition to identifying the direct effects of diabetes, recent studies have shown that the use of antidiabetic agents is an independent risk factor for fractures in diabetes (Bazelier et al., 2003; Zhu et al., 2014). Thiazolidinediones (TZDs) are reportedly associated with an increased fracture risk (Zhu et al., 2014). A study investigated the association between fracture risk and other antidiabetic agents, including dipeptidyl peptidase-4 (DPP4) (Schwartz, 2017). The results of Majumdar et al and the TECOS trial revealed that sitagliptin use is not associated with an increased fracture risk (Majumdar et al., 2016; Josse et al., 2017). Additionally, in a nationwide cohort study, Choi et al reported that DPP4 inhibitors in combination with metformin may confer protective effects against fractures (Choi et al., 2016). Thus, till now, the association between sitagliptin use and an increased fracture risk is either neural or protective. The association between days of sitagliptin and fracture risks remained unknown. Further, since sitagliptin is a second line agent for diabetes control and it is expensive compared to other antidiabetic agents. Considering relatively high fracture rates in diabetic population, it remained unknown whether the medical costs of sitagliptin users after fracture is higher or lower compared with non-sitagliptin users after fracture. Therefore, we conducted a large nationwide controlled cohort study in Taiwan to investigate the possible fracture risks of sitagliptin users and medical costs after fractures of sitagliptin users.

## Methods

### Data source

This retrospective cohort study with secondary data analysis was conducted using the Longitudinal Health Insurance Database (LHID) 2000, a subset of the National Health Insurance Research Database (NHIRD) of Taiwan. The NHIRD includes information on nearly 99% of the 23.74 million persons in Taiwan and is managed and released by the National Health Research Institutes (NHRI) (Database NHIR, 2015). Briefly, the LHID2000 was created by randomly selecting 1,000,000 enrollees from the 2000 Registry for Beneficiaries of the NHIRD. The LHID2000 has been confirmed by the NHRI to be representative of Taiwanese residents. Diseases in the LHID2000 are defined on the basis of the International Classification of Diseases, Ninth Revision, Clinical Modification (ICD-9-CM) codes.

### Data availability statement

The dataset used in this study is held by the Taiwan Ministry of Health and Welfare (MOHW). The Ministry of Health and Welfare must approve our application to access this data. Any researcher interested in accessing this dataset can submit an application form to the Ministry of Health and Welfare requesting access. Please contact the staff of MOHW (Email: stcarolwu@mohw.gov.tw) for further assistance. Taiwan Ministry of Health and Welfare Address: No.488, Sec. 6, Zhongxiao E. Rd., Nangang Dist., Taipei City 115, Taiwan (R.O.C.). Phone: +886-2-8590-6848. All relevant data are within the paper.

### Ethics statement

The NHIRD encrypts patient personal information to protect privacy and provides researchers with anonymous identification numbers associated with relevant claims information, including sex, date of birth, medical services received, and prescriptions. Therefore, patient consent is not required to access the NHIRD. This study was approved to fulfill the condition for exemption by the Institutional Review Board (IRB) of China Medical University (CMUH104-REC2-115-CR2). The IRB also specifically waived the consent requirement.

### Patients

Patients with type 2 diabetes (ICD-9-CM 250.x0 and 250.x2) aged 20 years and above between 2009 and 2012 were divided into 2 cohorts according to sitagliptin use. The sitagliptin cohort included patients who received sitagliptin therapy for at least 28 days, and the nonsitagliptin cohort comprised patients without any sitagliptin therapy. The date on which sitagliptin therapy was commenced was considered the index date. The index date for non-sitagliptin users was randomly appointed a month and day with the same index year of the matched sitagliptin users. Patients younger than 20 years, those having a history of fracture (ICD-9-CM 820–829), and those with incomplete age or sex information were excluded. Patients who received sitagliptin were matched (1:1 ratio) with those who did not receive sitagliptin therapy according to their propensity score (PS) through nearest neighbor matching, initially to the eighth digit and then as required to the first digit. Therefore, matches were first made within a caliper width of 0.0000001, and then the caliper width was increased for unmatched cases to 0.1. We reconsidered the matching criteria and performed a rematch (greedy algorithm) (Parsons, 2004). The PS was calculated using logistic regression to estimate the probability of treatment assignment, based on the baseline variables, namely the year of receiving sitagliptin therapy; age; sex; the adapted Diabetes Complications Severity Index (aDCSI); comorbidities of rheumatoid arthirits, osteoporosis, hypertension, hyperlipidemia, stroke, chronic obstructive pulmonary disease (COPD), cirrhosis, chronic kidney disease (CKD), depression, fibromyalgia, coronary artery disease, alcohol-related diseases, biliary stone, asthma, and peptic ulcer disease (PUD); and use of steroids, benzodiazepines (BZDs), TZDs (including pioglitazone, rosiglitazone), insulin, sulfonylureas (including cetohexamide, chlorpropamide, glibenclamide, glibornuride, gliclazide, glimepiride, glipizide, gliquidone, tolazamide, and tolbutamide), metformin, and other antidiabetic agents (including acarbose, exenatide, guar_gum, liraglutide, miglitol, mitiglinide, nateglinide, repaglinide). Additional sitagliptin users and non-sitagliptin users unmatched population were also showed in Table 1.

**Table 1:** Comparisons in demographic characteristics and comorbidities in type 2 diabetes mellitus patient with and without sitagliptin.

	**Unmatched population**					**Propensity Score Matched**				
	**Sitagliptin**					**Sitagliptin**				
	**Yes** ***N*** = **5,311**		**No** ***N*** = **18,080**			**Yes** ***N*** = **1,463**		**No** ***N*** = **1,463**		
	***n***	**%**	***n***	**%**	***p*-value**	***n***	**%**	***n***	**%**	***p*-value**
Age group					< 0.001					0.93
≤49	724	13.6	4019	22.2		201	13.7	204	13.9	
50-64	2,255	42.5	6460	35.7		578	39.5	568	38.8	
≥65	2,332	43.9	7601	42.0		684	46.8	691	47.2	
Age, year Mean (SD)^#^	63.0	12.4	60.9	13.8	< 0.001	63.5	12.7	63.5	12.5	0.97
Sex					0.23					0.79
Female	2,362	44.9	7940	43.9		636	43.5	629	43.0	
Male	2,929	55.2	10140	56.1		827	56.5	834	57.0	
Mean aDCSI score (SD)	0.36	2.18	0.40	1.20	0.11	0.37	0.74	0.30	1.53	0.09
Duration of diabetes	8.25	3.88	1.31	5.18	< 0.001	7.27	3.76	7.24	3.65	0.84
**COMORBIDITY**										
Rheumatoid arthritis	15	0.28	44	0.24	0.62	6	0.41	4	0.27	0.53
Osteoporosis	525	9.89	1471	8.14	< 0.001	140	9.57	159	10.9	0.25
Hypertension	4,318	81.3	11728	64.9	< 0.001	1176	80.4	1155	79.0	0.33
Hyperlipidemia	4,079	76.8	8094	44.8	< 0.001	1061	72.5	1051	71.8	0.68
Stroke	1,620	30.5	5921	32.8	0.002	460	31.4	478	32.7	0.48
COPD	1,036	19.5	3159	17.5	< 0.001	321	21.9	303	20.7	0.42
Cirrhosis	1,958	36.9	5345	29.6	< 0.001	552	37.7	559	38.2	0.79
CKD	595	11.2	900	4.98	< 0.001	158	10.8	148	10.1	0.55
Depression	441	8.30	956	5.29	< 0.001	135	9.23	128	8.75	0.65
Fibromyalgia	1,607	30.3	3060	16.9	< 0.001	421	28.8	442	30.2	0.39
Coronary artery disease	5,758	31.9	2394	45.1	< 0.001	599	40.9	596	40.7	0.91
Alcohol-related diseases	446	8.40	909	5.03	< 0.001	119	8.13	118	8.07	0.95
Biliary stone	255	4.80	465	2.57	< 0.001	67	4.58	66	4.51	0.93
Asthma	654	12.3	1730	9.57	< 0.001	190	13.0	176	12.0	0.43
PUD	3,574	67.3	9347	51.7	< 0.001	995	68.0	987	67.5	0.75
**MEDICATION**										
Steroid	5,106	96.1	15237	84.3	< 0.001	1396	95.4	1395	95.4	0.93
BZD	4,687	88.3	13677	75.7	< 0.001	1260	86.1	1262	86.3	0.91
TZD	2,883	54.3	1844	10.2	< 0.001	589	40.3	534	36.5	0.04
Insulin	3,333	62.8	5009	27.7	< 0.001	844	57.7	811	55.4	0.22
Sulfonylureas	5,030	94.7	10253	56.7	< 0.001	1336	91.3	1336	91.3	0.99
Metformin	5,157	97.1	9301	51.4	< 0.001	1375	94.0	1380	94.3	0.69
Other antidiabetic	3,368	63.4	2875	15.9	< 0.001	781	53.4	754	51.5	0.32
The number of antidiabetic agents					< 0.001					0.002
0	44	0.83	7333	40.6		31	2.12	44	3.01	
1-3	2,188	41.2	8250	45.6		774	52.9	792	54.1	
4-6	2,681	50.5	2370	13.1		571	39.0	581	39.7	
≥7	398	7.49	127	0.70		87	5.95	46	3.14	

Chi-Square Test,

# Student's t-test.

*aDCSI, adapted Diabetes Complication Severity Index*.

### Outcome measurement

All patients were followed from the index date to the incidence of fractures, withdrawal from the NHI program, or the end of 2013, whichever occurred first. Total outpatient and inpatient medical costs within 1-year period following fractures were also measured.

### Variables of interest

The mean numbers of days of sitagliptin treatment were divided into 3 categories by setting cutoff values on the basis of the first (110 days) and second quartiles (250 days). We evaluated diabetes severity according to the aDCSI, which was reported to be a useful tool for categorizing the severity of diabetic complications (Young et al., 2008). The progression of diabetes was defined as an annual increase in the aDCSI from the date of diagnosis to the end of follow-up. The four groups were defined based on quartiles. Four progression groups were defined on the basis of an annual increase in the score of less than 0.51, 0.51–1.00, 1.01–2.00, and more than 2.0. Different progression groups indicated slow, moderate, rapid, and very rapid progression.

### Statistical analysis

The sitagliptin and nonsitagliptin cohorts were unmatched and were matched according to the PS. To estimate the PS, a logistic regression model was used, in which the sitagliptin treatment status was regressed on the baseline characteristics listed in Table 1. We described and compared the distributions of the demographic variables, comorbidities (%), and medications (%) between the 2 cohorts by conducting chi-squared tests. The mean ages and standard deviations (SDs) were obtained and examined using Student's *t*-test. We calculated the incidence density of fractures according to person-years in unmached cohort. We determined the overall incidence as well as the incidence stratified by sex, age group, comorbidities, medications, and follow-up time for sitagliptin and PS matched nonsitagliptin cohorts. Univariable and multivariable Cox proportional hazards models were used for estimating the hazard ratios (HRs) and 95% confidence intervals (CIs) of fractures for the sitagliptin cohort relative to the unmatched nonsitagliptin cohort and sitagliptin cohort relative to the PS matched nonsitagliptin cohort. Variable found to be significant in the univariable analysis were further examined in the multivariable analysis. Further analysis was performed to evaluate the risk of diabetes progression from the date of diagnosis to the end of follow-up in the 4 progression groups to determine whether diabetes progression can predict fracture risk. We also assessed the joint effects of sitagliptin and other antidiabetic agents on fracture events. Data were analyzed with SAS (Version 9.3 for Windows; SAS Institute, Inc., Cary, NC, USA). All statistical tests were conducted at the 2-tailed significance level of 0.05.

## Results

**Figure 1:**
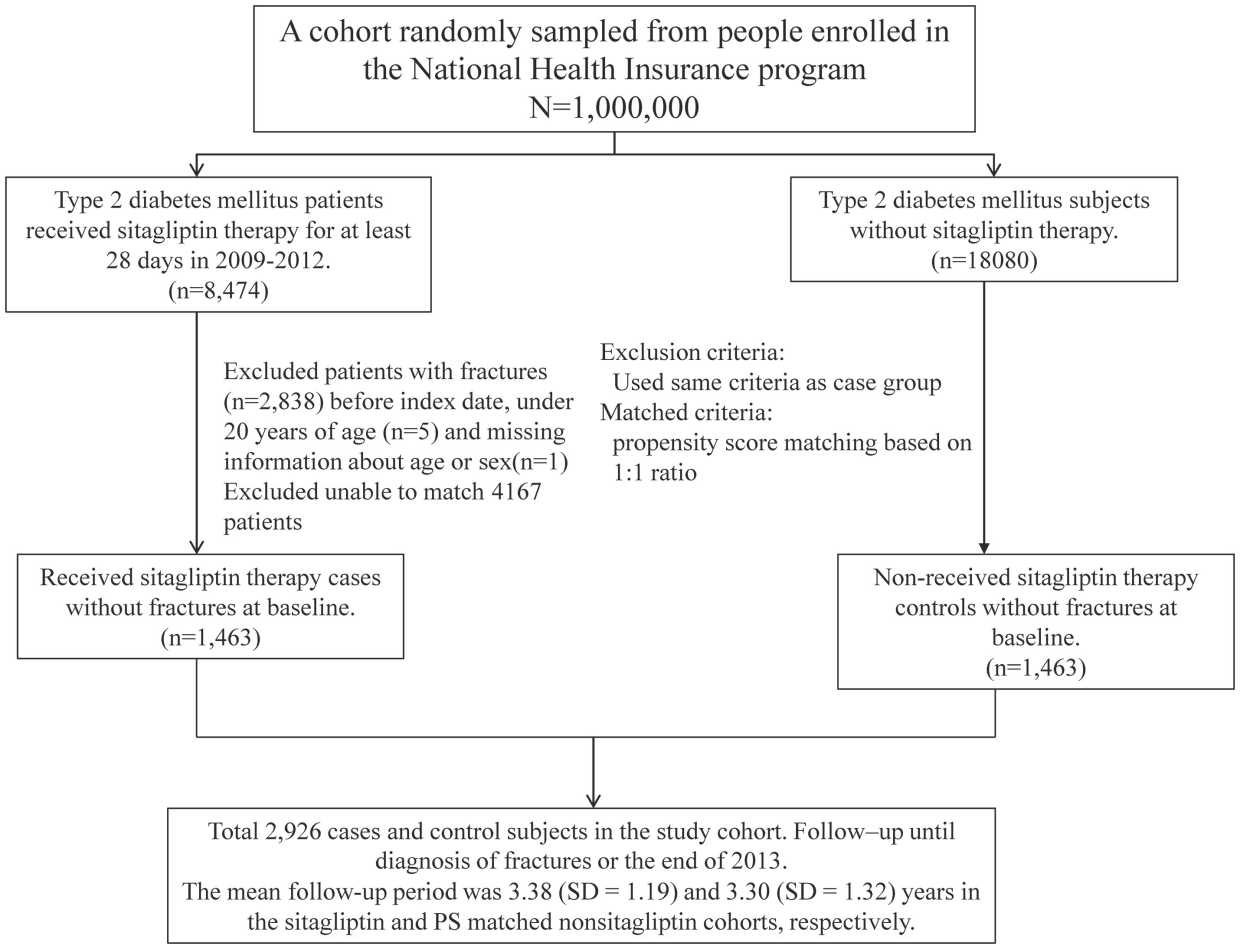
The enrolling process of this study.

We identified 5,311 sitagliptin users and 18 080 nonsitagliptin patients as the unmatched cohorts. In addition, 1,463 and 1,463 patients in the sitagliptin and nonsitagliptin cohorts according to the PS matched (Figure 1). The two unmatched cohorts were significantly difference in the baseline characteristics. The mean age of patients in the sitagliptin and PS matched nonsitagliptin cohorts was 63.5 (SD = 12.7) and 63.5 (SD = 12.5) years, respectively. Patients aged ≥65 years were 44.9 vs. 47.2% in sitagliptin and nonsitagliptin cohort. Both matched cohorts included more men than women (56.5 vs. 57.0%). The mean aDCSI was 0.37 ± 0.74 years in the sitagliptin cohort and 0.30 ± 1.53 years in the PS matched nonsitagliptin cohort. The major comorbidities and medications used in the 2 cohorts were hypertension (80.4 vs. 79.0%), hyperlipidemia (72.5 vs. 71.8%), PUD (68.0 vs. 67.5%), steroids (95.4 vs. 95.4%), sulfonylureas (91.3 vs. 91.3%), and metformin (94.0 vs. 94.3%). The mean follow-up period was 3.38 (*SD* = 1.19) and 3.30 (*SD* = 1.32) years in the sitagliptin and PS matched nonsitagliptin cohorts, respectively (data not shown). Most of the matched patients had the number of antidiabetic agents of 1–3 (52.9 vs. 54.1%).

Overall, the sitagliptin cohort had higher incidence density rates of fractures (44.6 per 1,000 person-years) than did the nonsitagliptin cohort (44.5 per 1,000 person-years), with a crude HR of 1.02 (95% CI = 0.85–1.24; Table 2). Multivariable Cox proportional hazards regression analysis for the risk of fracture revealed a nonsignificantly higher risk in the sitagliptin cohort (adjusted HR [aHR] = 1.01, 95% CI = 0.83–1.22) than in the nonsitagliptin cohort. The risk of fracture was not significantly higher in the unmatched sitagliptin cohort than in the unmatched nonsitagliptin cohort (aHR = 1.03, 95% CI = 0.93–1.14). After all stratification, the relative risk of fracture was not significantly higher in the sitagliptin cohort than in the PS matched nonsitagliptin cohort (Table 3).

**Table 2:** Comparison of incidence densities of fracture and hazard ratio between type 2 diabetes mellitus patient with and without sitagliptin by unmatched population and Propensity Score Matched.

	**Unmatched population**		**Propensity Score Matched**	
	**Sitagliptin**		**Sitagliptin**	
	**Yes *N* = 5,311**	**No *N* = 18080**	**Yes *N* = 1463**	**No *N* = 1463**
**FRACTURE**				
Event	607	4,500	221	215
Person-years	13,987	117,247	4,952	4,827
Rate^#^	43.4	38.4	44.6	44.5
Crude HR (95% CI)	1(Reference)	1.18(1.08, 1.29)^***^	1(Reference)	1.02(0.85, 1.24)
Adjusted HR (95% CI)^‡^	1(Reference)	1.03(0.93, 1.14)	1(Reference)^†^	1.01(0.83, 1.22)

Rate^#^, incidence rate, per 1,000 person-years; HR, relative hazard ratio;

† Variable found to be significant in the univariable analysis were further examined in the multivariable analysis for the matched sample.

**Comorbidity‡:** Patients with any one of the comorbidities (including osteoporosis, hypertension, hyperlipidemia, stroke, COPD, cirrhosis, CKD, depression, fibromyalgia, coronary artery disease, alcohol-related diseases, biliary stone, asthma, PUD) were classified as the comorbidity group.

*** *p < 0.001*.

**Table 3:** Comparison of incidence densities of fracture and hazard ratio between type 2 diabetes mellitus patient with and without sitagliptin mellitus by demographic characteristics and comorbidity.

	**Propensity Score Matched**							
	**Sitagliptin**							
	**Yes**			**No**				
	**Event**	**Person-years**	**Rate^#^**	**Event**	**Person-years**	**Rate^#^**	**Crude HR (95% confidence interval)**	**Adjusted HR^†^ (95% confidence interval)**
**SEX**								
Female	125	2,132	58.6	116	2,051	56.6	1.06(0.82, 1.37)	1.08(0.84, 1.40)
Male	96	2,820	34.0	99	2,776	35.7	0.97(0.73, 1.29)	0.95(0.72, 1.27)
**STRATIFY AGE**								
≤ 49	14	732	19.1	19	747	25.5	0.74(0.37, 1.47)	0.63(0.30, 1.32)
50-64	77	2,060	37.4	76	1,972	38.5	0.97(0.71, 1.34)	0.99(0.72, 1.37)
≥65	130	2,160	60.2	120	2,108	56.9	1.10(0.86, 1.42)	1.07(0.83, 1.38)
**COMORBIDITY**^‡^								
No	1	44	22.6	6	79	76.2	0.29(0.04, 2.45)	0.23(0.02, 3.19)
Yes	220	4,908	44.8	209	4,749	44.0	1.04(0.86, 1.26)	1.03(0.85, 1.24)
**MEDICATION**								
**Steroid** NO	8	227	35.3	5	239	20.9	1.59(0.52, 4.86)	2.68(0.57, 12.7)
Yes	213	4,725	45.1	210	4,588	45.8	1.01(0.83, 1.22)	1.00(0.82, 1.21)
**BZD**								
No	21	710	29.6	23	707	32.6	0.89(0.49, 1.61)	0.89(0.48, 1.65)
Yes	200	4,242	47.2	192	4,121	46.6	1.04(0.85, 1.27)	1.03(0.84, 1.26)
**TZD**								
No	126	2,822	44.6	126	3,007	41.9	1.06(0.83, 1.36)	1.06(0.82, 1.36)
Yes	95	2,130	44.6	89	1,821	48.9	0.96(0.72, 1.29)	0.92(0.68, 1.24)
**INSULIN**								
No	88	2,153	40.9	91	2,225	40.9	1.02(0.76, 1.37)	1.07(0.79, 1.45)
Yes	133	2,799	47.5	124	2,602	47.7	1.02(0.80, 1.31)	1.00(0.78, 1.29)
**SULFONYLUREAS**								
No	14	400	35.0	18	403	44.6	0.82(0.41, 1.64)	0.93(0.42, 2.05)
Yes	207	4,552	45.5	197	4,424	44.5	1.05(0.86, 1.27)	1.03(0.85, 1.26)
**METFORMIN**								
No	13	262	49.5	11	270	40.7	1.27(0.57, 2.84)	0.87(0.30, 2.58)
Yes	208	4,689	44.4	204	4,557	44.8	1.01(0.83, 1.23)	1.00(0.82, 1.22)
**OTHER ANTIDIABETIC**								
No	83	2,305	36.0	90	2,382	37.8	0.96(0.71, 1.30)	0.99(0.73, 1.34)
Yes	138	2,647	52.1	125	2,446	51.1	1.05(0.82, 1.34)	1.05(0.82, 1.34)
**FOLLOW-UP TIME**								
≤ 1 years	55	1,411	39.0	62	1,391	44.6	0.87(0.61, 1.26)	0.87(0.60, 1.24)
>1 years	166	3,541	46.9	153	3,437	44.5	1.09(0.87, 1.36)	1.07(0.86, 1.34)

Rate^#^, incidence rate, per 1,000 person-years; HR, relative hazard ratio;

† Variable found to be significant in the univariable analysis were further examined in the multivariable analysis.

**Comorbidity‡:** *Patients with any one of the comorbidities (including osteoporosis, hypertension, hyperlipidemia, stroke, COPD, cirrhosis, CKD, depression, fibromyalgia, coronary artery disease, alcohol-related diseases, biliary stone, asthma, PUD) were classified as the comorbidity group*.

The incidence and risk of fracture in the 2 cohorts were compared with respect to the aDCSI (Table 4). The incidence increased with the aDCSI in both cohorts. After stratification by the aDCSI, the relative risk of fracture was not higher in patients with an aDCSI of 0.00–0.50, 0.51–1.00, or >1.00.

**Table 4:** Comparison of incidence densities of fracture and hazard ratio between type 2 diabetes mellitus patient with and without sitagliptin by aDCSI change.

	**Propensity Score Matched**							
	**Sitagliptin**							
	**Yes**			**No**				
**Change in aDCSI Score per Year**	**Event**	**Person-years**	**Rate^#^**	**Event**	**Person-years**	**Rate^#^**	**HR (95% confidence interval)**	**Adjusted HR^†^ (95% confidence interval)**
0–0.50	161	3,674	43.8	172	3,657	47.0	0.95 (0.77, 1.18)	0.94 (0.76, 1.17)
0.51–1.00	30	938	32.0	27	886	30.5	1.10 (0.65, 1.86)	1.01 (0.58, 1.75)
>1.00	30	340	88.2	16	285	56.2	1.54 (0.84, 2.82)	1.73 (0.89, 3.37)

Rate^#^, incidence rate, per 1,000 person-years; HR, relative hazard ratio;

† *Variable found to be significant in the univariable analysis were further examined in the multivariable analysis*.

Table 5 shows that patients with the highest annual mean number of days of sitagliptin treatment (cutoff value: ≥250 days) no exhibited a higher risk of fracture compared with sitagliptin nonusers.

**Table 5:** Hazard ratio and 95% confidence intervals of fracture associated with annual mean the number of days, annual mean DDD (defined daily dose) or annual mean mg dose of sitagliptin exposure by Propensity Score Matched.

	***N***	**No. of Events**	**Rate^#^**	**Crude HR**	**95% confidence interval**	**Adjusted HR^†^**	**95% confidence interval**
Fracture	–	–	–	–	–	–	–
Non-use of sitagliptin	1,463	215	44.5	1	(Reference)	1	(Reference)
Sitagliptin^&^	–	–	–	–	–	–	–
< 110 days	365	50	38.8	0.89	(0.65, 1.21)	0.89	(0.65, 1.21)
110–250 days	369	46	35.3	0.81	(0.59, 1.12)	0.78	(0.57, 1.08)
≥250 days	729	125	53.0	1.22	(0.97, 1.52)	1.20	(0.96, 1.50)
p for trend				0.21	0.28

& The annual mean the number of days is partitioned in to 3 segments by fist quartile and second quartile.

Rate^#^, incidence rate, per 1,000 person-years; HR, relative hazard ratio;

† *Variable found to be significant in the univariable analysis were further examined in the multivariable analysis*.

We analyzed the joint effects of sitagliptin and other antidiabetic agents on fracture risk (Table 6). The risk of fracture was nonsignificantly higher in patients administered both sitagliptin and metformin (aHR = 0.94, 95% CI = 0.57–1.56) and those administered both sitagliptin and sulfonylureas (aHR = 0.94, 95% CI = 0.50–1.76) than in those not administered sitagliptin, metformin, or sulfonylureas. Moreover, the joint effects of sitagliptin plus TZDs and sitagliptin plus insulin on fracture risk were not significant.

**Table 6:** Cox proportional hazard regression analysis for the joint effect of Sitagliptin and medications on fracture risk by Propensity Score Matched.

**Variables**		**Event**	**Rate^#^**	**Adjusted HR^†^ (95% CI)**	***P*-value^&^**
Sitagliptin	TZD	–	–	–	0.49
No	No	126	41.9	1 (Reference)	–
Yes	No	89	48.9	1.08(0.81, 1.43)	–
No	Yes	126	44.6	1.07(0.81, 1.37)	–
Yes	Yes	95	44.6	1.00(0.76, 1.32)	–
Sitagliptin	Sulfonylureas	–	–	–	0.64
No	No	11	40.7	1 (Reference)	–
Yes	No	204	44.8	0.94(0.50, 1.76)	–
No	Yes	13	49.5	1.18(0.53, 2.66)	–
Yes	Yes	208	44.4	0.94(0.50, 1.76)	–
Sitagliptin	Meformin	–	–	–	0.47
No	No	18	44.6	1 (Reference)	–
Yes	No	197	44.5	0.91(0.55, 1.50)	–
No	Yes	14	35.0	0.71(0.35, 1.43)	–
Yes	Yes	207	45.5	0.94(0.57, 1.56)	–
Sitagliptin	Insulin	–	–	–	0.99
No	No	91	40.9	1 (Reference)	–
Yes	No	124	47.7	1.07(0.81, 1.42)	–
No	Yes	88	40.9	1.02(0.76, 1.37)	–
Yes	Yes	133	47.5	1.07(0.81, 1.41)	–

Rate^#^, incidence rate, per 1,000 person-years;

† Variable found to be significant in the univariable analysis were further examined in the multivariable analysis.

& *P-value for interaction*.

The average one-year medical cost after a fracture was 7,287 (*SD* = 9881.1) US dollars in sitagliptin users and 200.2 (*SD* = 428.4) US dollars in non-sitagliptin users (*p* < 0.001). The average one-year medical costs of sitaglitpin users without fracture was 120.2(*SD* = 213.5) US dollars.

## Discussion

In this large nationwide representative cohort of insured patients with type 2 diabetes, longer sitagliptin use, and a mean treatment duration ≥250 days were associated with an increased fracture risk. Furthermore, our data revealed that sitagliptin had no interaction with TZDs, sulfonylureas, insulin, and metformin in increasing the risk of fracture. The progression of diabetes, defined as an annual increase in the aDCSI, although high, was not significantly related to fracture risk.

It is biologically plausible that sitagliptin promotes skeletal muscle regeneration. In a diabetic rat model, Glorie et al showed that sitagliptin could attenuate bone loss and increase bone strength (Glorie et al., 2014). Clinical evidence is lacking on similar bone protection effects of sitagliptin. Several trials and cohort studies on sitagliptin have yielded neutral results (Driessen et al., 2014; Majumdar et al., 2016; Josse et al., 2017). The results of Majumdar et al. (2016), and the TECOS trial (Josse et al., 2017) revealed that sitagliptin is not associated with fracture in type 2 diabetes. In our study, sitagliptin was associated with an increased fracture risk in a specific group of patients while a mean treatment duration ≥250 days. Several possible explanations account for such an inconsistency between the previous (Majumdar et al., 2016; Josse et al., 2017) and present findings. First, the median follow-up duration was different in the study of Majumdar et al. (2016) (2 years) and the TECOS trial (3 years). Second, the analyzed clinical variables were different. Although all 3 studies incorporated age, antidiabetic agents, CAD, and PAD as factors influencing fractures, the current study also identified retinopathy, metabolic disorders, nephropathy, and neuropathy as clinical variables and weighed these variables by using the DCSI (Young et al., 2008). Further, the unit for evaluating the effect of sitaglitpin on fracture risks is different. This study adapted mean duration of follow up which confered two clinical variables, including duration of follow up and compliance. Thus, we believe that our study design could provide a more objective evaluation of the association of sitagliptin with fracture risk. It is interesting to note that cut point of 250 days represents either fracture risk or fracture protection of sitaglitpin. That would be possible reason why previous studies showed the effects of fractures either neutral or protective.

Finally, the population in the study of Majumdar et al. (2016) study was relatively young (median age: 52 years), and diabetes control in the TECOS trial (Josse et al., 2017) was reasonable; these factors are considered to be associated with low fracture risk and might account for a lower incidence of fracture in those study populations. In this study, we used LHID, a subset of the National Health Insurance Research Database (NHIRD) of Taiwan, which encompasses medical information of one million insured people extracted from NHIRD. Further, we used propensity matching strategy to match the study cohort and control cohort. We also provide demographic information of unmatched population which would help understand the difference in matched and unmatched population. The advantages of this database and analyzing strategy are that it enables longitudinal follow up of each insured person and nation-based investigation to minimize possible surveillance bias.

The pathophysiology of diabetes in association with fractures is complex; diabetes duration, vision, falls, neuropathy, underlying nephropathy, and concomitant antidiabetic agents are possible contributing factors. Thus, it is difficult to assess the effects of individual antidiabetic agents on fracture risk in type 2 diabetes. This large, retrospective cohort study could address most of these inherent problems by using propensity-matching methods and the DCSI (Young et al., 2008). The aDCSI enables adjusting for the severity of diabetes comorbidities. Thus, this study provides a relatively homogenous baseline for the comparing the risk of fracture in diabetes.

This study found that the average 1-year medical costs after fracture of sitagliptin users are higher than non-sitagliptin diabetic users. The possible reason might be the characteristics of sitagliptin users. Since sitagliptin is 2nd line agent for diabetes control, people who needed sitagliptin prescription would be worse diabetic control. Thus, sitagliptin users might cost much medical costs compared with non-sitagliptin users. Whether sitagliptin had direct effects on causing more complex fracture and difficult healing would need further studies and investigations.

However, the study has some limitations. First, information about individual risk factors for osteoporosis, including smoking, family history, vitamin D consumption, sun exposure, body frame size, exercise habits, alcohol use, and caffeine use, medical compliance, were unavailable in the database. Second, the study was conducted based on ICD-9-CM codes recorded in the NHIRD; thus, detailed information about the levels of hemoglobin A1c, testosterone, and estrogen were unavailable. However, we matched patients according to the aDCSI and hypoglycemic episodes to minimize bias. Third, we had no information about the bone mineral density (BMD) of each individual, including data on dual energy X-ray absorptiometry. However, patients with type 2 diabetes are reported to have a higher BMD than those without diabetes (Ma et al., 2012). Thus, the BMD might not be an appropriate indicator of fracture in diabetes. Fourth, most patients in this study were Taiwanese; thus, our findings should be cautiously applied to other populations. Finally, low evens and CKD patients might have lower duration of medications use would be possible limitations in this study, but this would not cause major bias of the results.

This study revealed that the sitagliptin for ≥ 250 days had a higher risk of fracture, irrespective of the aDCSI. The average one-year medical costs after fracture of sitagliptin users are significantly higher than non-sitagliptin diabetic users. This finding prompts clinical awareness of potential fracture risk in patients with diabetes receiving sitagliptin treatment, rather than discouraging the prescription of sitagliptin. In addition, we provided clear joint effect of sitagliptin with other anti-diabetic agents and medical costs after fracture in sitaglitpin users. From payer and societal perspective, it might be needed to reconsider the rules of using sitaglitpin in diabetes. Future studies with longer follow-up periods are required to validate our findings.

## Author contributions

S-YL and C-HK: conceptualization; C-LL and C-HK: methodology; C-LL, C-YH, and C-HK: software; S-YL, W-HH, C-CL, C-LL, C-HT, H-CY, C-YH, and C-HK: validation; S-YL, W-HH, C-CL, C-LL, C-HT, H-CY, C-YH, and C-HK: formal analysis; C-LL and C-HK: investigation; C-LL and C-HK: resources; S-YL, W-HH, C-CL, C-LL, C-HT, H-CY, C-YH, and C-HK: data curation; S-YL, W-HH, C-CL, C-LL, C-HT, H-CY, C-YH, and C-HK: writing (original draft preparation); S-YL, W-HH, C-CL, C-LL, C-HT, H-CY, C-YH, and C-HK: writing (review and editing); S-YL, W-HH, C-CL, C-LL, C-HT, H-CY, C-YH, and C-HK: Visualization; C-HK: supervision; C-HK: project administration; C-HK: funding acquisition.

### Conflict of interest statement

The authors declare that the research was conducted in the absence of any commercial or financial relationships that could be construed as a potential conflict of interest.
